# A Review of the Applicability of Current Green Practices in Healthcare Facilities

**DOI:** 10.34172/ijhpm.2023.6947

**Published:** 2023-06-06

**Authors:** Ana Luísa Soares, Sandra C. Buttigieg, Bartosz Bak, Sonya McFadden, Ciara Hughes, Patricia McClure, Jose Guilherme Couto, Isabel Bravo

**Affiliations:** ^1^Medical Physics Service, Portuguese Oncology Institute of Porto, Porto, Portugal; ^2^Department of Health Systems Management and Leadership, Faculty of Health Sciences, University of Malta, Msida, Malta; ^3^Radiotherapy Department II, Greater Poland Cancer Center, Poznan, Poland; ^4^Department of Electroradiology, University of Medical Science, Poznan, Poland; ^5^Faculty of Life and Health Sciences, Ulster University, Newtownabbey, UK; ^6^Radiography Department, Faculty of Health Sciences, University of Malta, Msida, Malta; ^7^Medical Physics and Radiobiology Group, Research Center (IPOP), Portuguese Oncology Institute of Porto, Porto, Portugal

**Keywords:** Circular Economy, Carbon Footprint, Healthcare Waste, Healthcare Management, Hospital Sustainability

## Abstract

**Background:** Circular economy (CE) has raised great interest as a concept and as a development model worldwide. This concept aims to provide a substitute for the linear economic model, which was based on production and consumption, continuous growth, and resources depletion. CE allows a greener economy with sustainable development and promotes more balanced societies. The healthcare sector is a major contributor to the climate crisis, with a carbon footprint representing 4.4% of global net emissions. It is thus essential to rethink the applicability of CE in healthcare.

**Methods:** We conducted a scoping review guided by the Arksey and O’Malley methodological framework and utilised PRISMA-ScR (Preferred Reporting Items for Systematic reviews and Meta-Analyses extension for Scoping Reviews) checklist. A systematic search from MEDLINE complete, SCOPUS, and Web of Science databases published between 1992 and 2022.

**Results:** Through database searching a total of 1018 records were identified and 475 duplicates were removed. From the total search, 543 articles were screened by title/abstract according to the inclusion and exclusion criteria. After screening, 38 full-text articles were selected and assessed for eligibility. Forty-seven additional records were also identified through other sources and screened for eligibility. Other sources included: 12 articles from snowballing of previous papers; 9 articles following peer-reviewers suggestions; 19 reports from relevant organisations in CE and healthcare; two webpage, and one book.

**Conclusion:** Specific areas were identified where hospitals could reduce their greenhouse gas (GHG) emissions and consequently their negative environmental impact, namely through waste management, energy, water, transportation/travel, hospital design, food optimisation, green procurement, and behaviour. Also, lack of staff awareness and knowledge of the environmental impact of healthcare, and hospitals sustainability were identified as major contributors.

## Background

 In the last few years, the increase in global population, scarcity of natural resources and increased waste have drawn the attention of policy-makers to continuously evolving challenges and major concerns in environmental sustainability. An extensive change in multisectoral policies with potential environmental impact is needed. For example, strict evaluation of current material production and population consumption patterns is required to reduce the resource depletion occurring worldwide.^1,2^

 A linear economy is an economic model based on converting natural resources and raw materials into waste in two approaches: firstly, extracting natural assets from the environment, and secondly through reducing capital value caused by pollution and waste. Pollution can also occur during resource extraction.^3^ Continuous economic growth, production and consumption, as well as increasing resource output represent the basis of the linear economic model.^1,2^ Linear economy goes against the current needs for environmental sustainability of the planet and the population’s well-being.^3^

 In contrast, circular economy (CE) is a model where the economy has no net effect on the environment, repairing the damage done in resource acquisition and creating less waste during the production procedure and in the product’s life cycle.^3^ Indeed, the global debate on the paradigm shift from a linear economic model to a CE has increased over the last decade. It is becoming evident that CE allows a greener economy with sustainable development and promotes a better balance between society, environment and economy.^1,2,4^

 CE aims at efficiently using resources through waste minimisation, lasting value of resources, reduced raw and scarce materials, and closed loops of products within the limits of environmental, social and economic benefits.^3^ CE is defined as an economic approach based on reduction, reuse, recovery and recyclability (3Rs: reduce, reuse, recycle) of materials, resources and energy.^5^ This strategy enhances the value and lifecycle of the products, materials and resources.^1,5^

 Therefore, the concept of CE encompasses two other concepts of sustainability: reducing resource use (such as materials, water, and energy) and reducing waste and its adequate management (through reuse or recycling). As such, these concepts were evaluated in this study.

 Another essential sustainability concept is the reduction of the carbon footprint and greenhouse gas (GHG) emissions. Even though it may be interpreted as a separate concept from CE, CE naturally reduces GHG emissions since it requires reducing and reusing resources, decreasing the production of goods, which leads to a decrease in GHG emissions of up to 70%.^6,7^ In addition, if we look at the fuels that produce the GHG emission as “resources,” limiting the use of these resources (R – Reduce), then it can be interpreted that the reduction in GHG emissions is included in the CE concept. In this study, the GHG reduction in healthcare was also evaluated.

 CE represents the most recent attempt to sustainably integrate economic activity and environmental well-being while decoupling economic growth from the negative impact of resource depletion and environmental damage.^3,4,8^ It is envisaged that a shift to a CE model would increase the workforce by 4% as part of the ambitious low-carbon economy goal.^7^ CE is, therefore, an essential element in achieving “environmentally sustainable healthcare facilities,” which is defined by the World Health Organization (WHO) as “those that improve, maintain or restore health, while minimising negative impacts on the environment and leveraging opportunities to restore and improve it.”^9^

 The rationale for this study is that the healthcare sector is considered one of the largest industries worldwide and is a significant contributor to the climate crisis, with a carbon footprint corresponding to 4.4% of global net emissions.^10-12^

 Therefore, the aim of this study is to determine the current application of the concept of CE within hospital practice. This scoping review also addressed other sustainability concepts parallel to CE, such as resource usage, waste management, and GHG emissions. The following research questions were formulated to guide this scoping review:

1. What is the current status of CE implementation within the European Union (EU)?
2. What is the applicability of CE in hospitals?
3. How can the application of CE to healthcare be expanded or improved?

## Materials and Methods

###  Search Strategy

 The scoping review was guided by the Arksey and O’Malley methodological framework and utilised PRISMA-ScR (Preferred Reporting Items for Systematic reviews and Meta-Analyses extension for Scoping Reviews) checklist.^13^ A systematic search from MEDLINE complete, SCOPUS, and Web of Science databases published between 1992 and 2022. The search strategy was conducted in line with the research questions, using selected keywords and their synonyms. In the SCOPUS database, the search was only performed by abstract and title.

 The keywords used were: (“circular economy” OR “carbon footprint”) AND (“hospital” OR “healthcare”).

###  Inclusion and Exclusion Criteria

 The selection of published studies is represented in the PRISMA flow diagram, as shown in Figure. The selection of the publications was based on the inclusion and exclusion criteria as described in Table 1. After identifying articles from the selected databases, snowballing was performed. An additional search was carried out, including grey literature, to increase the search scope. Articles that discuss economic, medical-economic or financial issues regarding the impact of CE in healthcare were excluded, as they were not the focus of this review.

**Figure:**
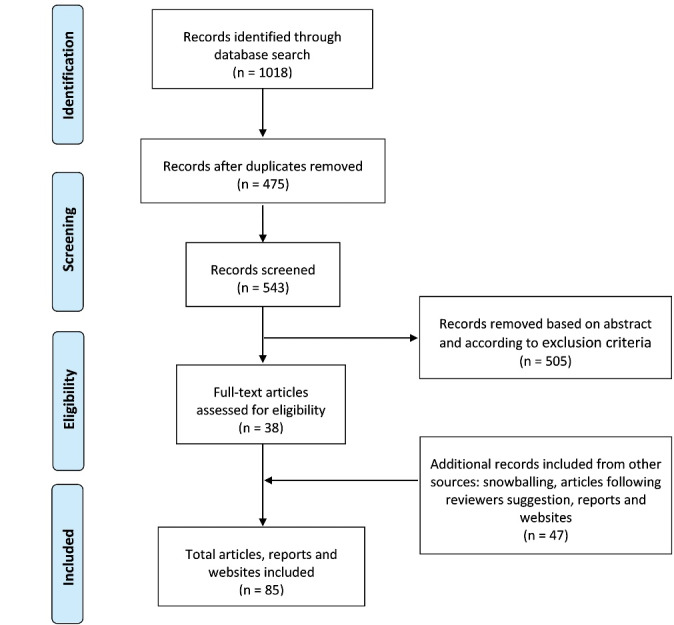
PRISMA Flow Diagram. Abbreviation: PRISMA, Preferred Reporting Items for Systematic Reviews and Meta-Analysis.

**Table 1:** Inclusion and Exclusion Criteria for Published Literature

**Inclusion Criteria**	**Exclusion Criteria**	**Main Justification**
Articles that are accessible on Medline complete, PubMed, Scopus*, and Web of Science.		These databases include both publications related to healthcare and sustainability.
Grey literature.		Grey literature was included to increase the search range (WHO, European Commission, Joint Research Centre – European Commission, United Nations, Centre for European Policy Studies Energy Climate House, Health Care Without Harm, Ellen MacArthur Foundation, European Environment Agency, and Foundation for Science and Technology).
Publications from 1992 to October 2022.		Recent articles on the CE are of interest to this review, since it focuses on current applications.
Publications that discuss the applicability of CE in healthcare, or their synonyms.	Non-specific to CE in healthcare. Economic, medical-economic or cost- effectiveness analyses. Letter to editor. Comments or opinion pieces.	Economic, medical-economic or financial impact of CE in healthcare was not the focus of this review. This study intended to focus on “research literature” which is supported by robust data collection, rather than “practice literature” as defined by Wallace and Wray.^14^

Abbreviations: WHO, World Health Organization; CE, circular economy. * Keywords searched on Abstract + Title only.

 This study did not include a formal critical appraisal of the literature since this scoping review aimed to gather all published work about the implementation of CE and GHG emissions in healthcare. As mentioned in Table 1, letters to the editor, comments and opinion pieces were not considered since they are not supported by robust data collections.

## Results

 Through database searching a total of 1018 records were identified and 475 duplicates were removed. From the total search, 543 articles were screened by title/abstract according to the inclusion and exclusion criteria. After screening, 38 full-text articles were selected and assessed for eligibility. Forty-seven additional records were also identified through other sources and screened for eligibility. Other sources included: 12 articles from snowballing of previous papers; 9 articles following peer-reviewers suggestion; 19 reports from relevant organisations in the area of CE and healthcare (WHO, European Commission, United Nations, Centre for European Policy Studies Energy Climate House, Health Care Without Harm Arup, Ellen MacArthur Foundation, and Foundation for Science and Technology; Circle Economy; Ministry of Environment of Brazil). Seven websites were also included (European Commission, Joint Research Centre – European Commission, United Nations, European Environment Agency, and WHO; Ministry of Health, Welfare and Sport of the Netherlands; Economist impact) (see Figure) and one book (*Critical reading and writing for postgraduates*).

 A summary of the articles included in this review regarding the type of study subject investigated, the relationship between behaviour/engagement and carbon footprint or environmental sustainability is provided in Table 2. It also presents which articles studied other subjects in addition to healthcare waste management and energy within hospitals. Table 2 further organises the articles according to the scope of the publication.

**Table 2:** Environmental Sustainability Wthin Hospitals

**Scope**	**Authors**	**Year**	**Country of Origin**	**Type of Study**	**Types of Waste/Waste Segregation or Healthcare Waste Management**	**Energy**	**Behaviour or Engagement and Environment Sustainability**	**Environmental Sustainability in Hospitals, Excluding Waste Management and Energy**
1. Several subjects	McGain and Naylor^15^	2014	Australia	Systematic review and research agenda	Yes	Yes	Yes	Yes
	Pencheon^16^	2015	UK	Mini-Symposium	Yes	Yes	Yes	Yes
	Tomson^17^	2015	UK	Opinion article	Yes	Yes	Yes	Yes
	Weimann and Patel^18^	2016	South Africa	Research paper	Yes	Yes	Yes	Yes
	Ryan-Fogarty et al^19^	2016	Ireland	Research article	Yes	Yes	Yes	Yes
	Langstaff and Brozozowski^20^	2017	Canada	Research article	Yes	Yes	Yes	Yes
	Bharara et al^21^	2018	India	Original article	Yes	Yes	Yes	Yes
	Voudrias^22^	2018	Greece	Editorial	Yes	-	-	Yes
	Aslan and Yıldız^23^	2019	Turkey	Original article	Yes	Yes	Yes	Yes
	Salas et al^24^	2020	USA, UK	Analysis	Yes	Yes	Yes	Yes
	Blum et al^25^	2020	USA	Research article	Yes	Yes	Yes	Yes
	Sherman et al^26^	2020	USA	Narrative review	Yes	Yes	Yes	Yes
2. Waste management	Muñoz^27^	2012	USA	Opinion article	Yes	-	Yes	-
	Manzi et al^28^	2014	England	Original research	Yes	-	Yes	-
	Ali et al^29^	2016	Pakistan	Case study	Yes	Yes	-	-
	Ali et al^30^	2017	China and Pakistan	Mini-review	Yes	-	Yes	-
	Capoor and Bhowmik^31^	2017	India	Research article	Yes	Yes	Yes	-
	Barbosa and Mol^32^	2018	Brazil	Research article	Yes	-	-	-
	Zamparas et al^33^	2019	Greece	Research article	Yes	Yes	Yes	-
	Ferronato et al^34^	2020	Italy	Research article	Yes	-	-	-
	Rizan et al^35^	2020	UK	Research article	Yes	Yes	-	Yes
3. Green building	García-Sanz-Calcedo et al^36^	2020	Spain and Portugal	Research article	-	Yes	-	Yes
	Sahamir et^37^	2019	Malaysia	Research article	-	Yes	-	Yes
	Prada et al^38^	2020	Romania and France	Research article	-	Yes	-	Yes
4. Travel transportation telemedicine	Wootton et al^39^	2010	UK	Research article	-	-	-	Yes
	Oliveira et al^40^	2013	UK, Portugal	Original Research	-	-	-	Yes
	Holmner et al^41^	2014	Sweden, USA, Indonesia	Research article	Yes	Yes	Yes	Yes
	Dullet et al^42^	2017	USA and Australia	Research article	-	-	-	Yes
	Vidal-Alaball et al^43^	2019	Spain	Research article	-	-	-	Yes
	Purohit et al^44^	2021	UK	Research article	-	Yes	-	Yes
	Sánchez-Barroso et al^45^	2021	Spain	Research article	-	Yes	-	Yes

- Scope 1 includes articles which explored hospital sustainability, with more than two important subjects, excluded waste management and energy, regarding environmental-friendly hospitals.
- Scope 2 lists articles which described healthcare waste management within hospitals, where the common themes were types of waste, waste streams, waste segregation and correct handling and disposal.
- Scope 3 covers the topic of green building, with research focused on energy efficiency and energy spending in hospital construction.
- Scope 4 includes articles which investigated travel/transportation and telemedicine: the articles explore the impact of travel to hospital and the potential of telemedicine.

Table 2 includes thirty-one articles and the common theme among them is environmental sustainability within hospitals. In the remainder articles a relationship with the presented scopes was not found.

 Scope 1 and 2 present the majority of the articles. Nevertheless, articles with scopes 3 and 4, provide other specific and important subjects regarding hospitals’ sustainability.

Table 2 indicates that waste management and energy have been the subjects most often studied regarding sustainability within hospitals. Furthermore, the energy subject is transversal to all scopes. Other subjects regarding sustainability in hospitals found in scope 1, directly related to CE, were food optimisation, green procurement/supply chain, education, and water consumption/efficiency, as it is shown is the last column of Table 2.

 An important aspect referred in most articles, is the relationship between behavior/engagement and the environmental sustainability. This was mainly included in paper under scope 1.

 Regarding geographical spread, this table presents eight articles from Europe, six from the United States and eight from the United Kingdom. No relationship was found between the scope of the articles and the country of origin. The most common type of study is ‘*Research article.*’ Table 2 also reveals that there has been an increase in the number of publications per year since 2014, and with an even more significant increase in 2019 and 2020.

Table 3 focused on articles that evaluated the carbon footprint of the healthcare systems in different countries.^12,46-52^ One article reported on carbon footprint comparison of carbon dioxide emissions for the healthcare sector across all Organisation for Economic Co-operation and Development (OECD) countries (except Chile), China, and India.^12^ A significant number of publications involved with environmental sustainability within hospitals were from the United Kingdom (eight) and Europe (eight), followed by the United States (six).

**Table 3:** Evaluation of the Carbon Footprint in Healthcare System

**Authors**	**Year**	**Country**	**Type of study**	**LCA**	**Type of Healthcare System**	**Context of the Study**	**Healthcare GHG Emissions or Carbon Footprint**
Eckelman and Sherman^46^	2016	USA	Research paper	Yes	Publicly financed: Medicare, Medicaid; Private insurance	Publicly financed: Private insurance	GHG emissions 10%
Malik et al^47^	2018	Australia	Research paper	Yes	Public hospitals, private hospitals	Public hospitals, private hospitals, primary healthcare, dental services, community healthcare	Carbon footprint/CO_2 _emissions 7% of total
Eckelman et al^49^	2018	Canada	Research paper	Yes	Mixed system: predominatly public hospitals	Public hospitals, private hospitals, primary healthcare, pharmaceuticals	Carbon footprint/GHG emissions 4.6% of total
Wu^48^	2019	China	Research paper	Yes	Mixed system: predominatly public hospitals	Public hospitals, private hospitals, community healthcare, public health, pharmaceuticals	Carbon footprint/GHG emissions 2.7% of total
Pichler et al^12^	2019	Germany, Vienna, Berlin	Environmental research letter	N/A	Mixed system	Hospitals, ambulatory healthcare, preventive/long-term healthcare	Healthcare on average accounts for 5% of the national carbon footprint
Alshqaqeeq et al^52^	2020	USA	Full length article	N/A	Carbon footprint in the 12 hospital service or department categories		Detailed description in the article
Nansai et al^50^	2020	Japan	Full length article	N/A	Public hospitals, mandatory health insurance coverage	Medical services, nursing services, health and hygiene, pharmaceuticals	Carbon footprint/GHG emissions 4.6% of total
Weisz et al^51^	2020	Austria	Full length article	N/A	Public funded universal healthcare, Private insurance	Hospitals, ambulatory healthcare, preventive/long-term healthcare, pharmaceuticals	National carbon footprint 7%

Abbreviations: LCA, life cycle assessment; GHG, greenhouse gas; N/A, not available.

 A lack of healthcare carbon footprint evaluation per county has been identified, as only seven articles reported the national healthcare GHG emissions/carbon footprint and one article regarding carbon footprint comparison of carbon dioxide emissions of the healthcare sector across all OECD countries (except Chile), China and India (Table 3). A common point between articles evaluating healthcare carbon footprint is the use of the life cycle assessment (LCA) evaluation as a valuable tool to measure environmental impact. Of all publications identified in the literature search, only eight discuss healthcare GHG emissions and carbon footprint (Table 3). Regarding hospitals or healthcare systems, we only found GHG emission or carbon footprint contributions from the United States, Australia, Canada, China, Japan, and Austria healthcare system, with two articles from the United States.

## Overview of the CE Implementation in the European Union

 Lack of familiarity and fear of the unknown may explain why the CE concept has been slow to gain attention worldwide.^7^ Being considereda holistic concept, it clashes with the fundamentals of scholars, corporations and administrations that prioritise the increase of gross domestic product over the creation of wealth through preserving scarce resources.^7^ However, CE’s applications to modern economic and industrial processes are becoming increasingly relevant. Since the 1970s, volatility in the global economy also raised pressure in the environment due to resource depletion.^4,8^ Despite the efforts aimed at incorporating sustainability into organisations, this is still under-researched. Also, sustainability changes in planning must also address technical, managerial, organisational issues, as well as the organisation’s stakeholders.^53^

 CE implementation is still in the initial phases, concentrated on recycling instead of reusing and sustainable consumption. However, secondary raw materials start to compete with virgin raw materials when technically possible and economically feasible. This makes CE promising as secondary goods could reduce the use of scarce primary materials.^1,5,7^

 In Europe, CE’s implementation primarily emerged through waste management policies aiming at highlighting the recycling principle.^54^ From a policy perspective, in 2014, the European Parliament adopted the communication from the European Commission (EC), ‘*Towards a Circular Economy: a zero waste programme for Europe.’* The intention was to boost recycling, prevent loss of valuable materials, and create employment and sustainable economic development by promoting new business models, eco-design, and industrial symbiosis, leading to a low-carbon society and reduced environmental impact.^3,55^

 At the EU level, the European Commission described the concept in its Communication ‘*Closing the loop – An EU Action Plan for circular economy*, part of the *Circular Economy Package.’*^56^ Accordingly, in 2015, the Commission adopted an ambitious CE Package to stimulate Europe’s transition towards CE. The EU demonstrated to economic operators that it was using all means to ensure economic transformation. New business opportunities would therefore improve global competitiveness, as well as also encouraging long term economic growth and job creation.^56^

 At the national level, countries implemented CE and other sustainability concepts at different levels.^57^ An example is the Dutch Green Deal on Sustainable Healthcare, which includes the circularity of materials (reduce, reuse and recycle medical waste), reduction of GHG emissions and waste.^58^

 The EC sees the transition to CE as a process that requires substantial changes in each step of the value chain and society.^56^ This model became of paramount importance for the EU’s commitment to a sustainable, low-carbon and competitive economy.^2,56^ This will be achieved to CE processes already discussed before, including preservation of raw materials, reduction in waste, improvement in products’ overall lifecycle, increase in innovation and efficiency of production and consumption, promotion of energy savings and reduction in GHG.^56^

 The EU policies are scientifically and technically supported by the Joint Research Centre, a science EC’s centre group.^59^ It aims to accomplish an efficient use of resources, such as those linked to recycling, waste management and efficiency standards or best available methods for production.^5,59^ According to the Joint Research Centre, the purpose of EU policy has been to promote CE specific subjects, namely: material-efficient products; best available techniques for sustainable production; environmental technology verification; end of waste criteria; ecolabel, green public procurement, eco-design and energy labelling; best environmental management practice; and sustainable use of resources (water, soil, raw materials, with special importance to minerals). These include energy efficiency, renewable energy, and natural resources.^5,59^ The EU recognises that raw materials will have a fundamental role in the production processes within CE, ensuring the security of supply and decreasing price volatility.^5^

 The CE package also includes actions focused on market barriers, in specific sectors or materials streams. These include mineral resources, plastic, food residues, construction waste, raw/virgin material, biomass, as well as measures in innovation and investment. The main objective is to focus on action levels where the EU could add value and make a difference in the field.^5^

 In 2011, the EC issued guidelines towards a competitive low-carbon economy in 2050. Accordingly, EU governments have adopted different strategies to implement and monitor emissions, with an ambitious programme to achieve zero emissions by 2050 to meet the 1.5˚C target set out in the Paris Agreement. The EU’s target to reduce GHG emissions inside the EU by at least 40% by 2030 and the ongoing implementation of a coherent set of climate and energy policies to achieve this is underlined in the 2050 roadmap.^60,61^

 Despite the importance that the EU gives to the implementation of CE and reduction of GHG, only 6 of the 27 EU countries performed climate change and health vulnerability and adaptation assessments (V&As) in 2015,^62^ however, many more V&As have been published by the WHO^57^ since then showing a tendency to give more importance to the issue in recent times. Which evaluate the main health vulnerabilities in their countries due to climate change. The main recommendation of the V&As at this stage is for healthcare facilities to make sustainable commitments regarding their footprint,^62,63^ with no reference to other CE concepts. This shows that there is still data missing and the importance of keeping collecting data to better inform decisions in the future.

 National Adaptation Plans offer a sector-specific report which includes the health sector. However, when they exist, they tend to focus on the impact of climate-change in the health and healthcare, rather than the sustainability of healthcare system and facilities.^64^ Other relevant reports are the WHO hospital safety index, which evaluates the readiness and resilience of healthcare facilities facing large-scale disasters and emergencies, which may increase with climate change.^65^ Like these, many climate-change reports mention the health impacts and often (rightly so) portrait healthcare as one of the “victims” of the climate-change; however, this study focus on the healthcare system as a contributor to the climate crisis and how to minimise this contribution.

## Applicability of Circular Economy in Healthcare

 The healthcare sector is one of the largest service sectors worldwide^13^: It is the fifth largest contributor to planetary pollution^66^ and the EU health sector, for example, accounts for 10% of gross domestic product, 15% of public expenditure and 8% of the EU’s workforce and has a high potential for innovation and growth.^22,33^ However, healthcare expenditures have overtaken economic growth, driven by an ageing population, life-styled related diseases, and advances in medical technology and treatments. As such, to honour its commitment to first “do no harm,” the health sector is responsible for measuring and mitigating the environmental impact associated with healthcare. People who are harmed by the environmental footprint of healthcare often live far away from the healthcare provided, so health professionals have both an ethical and a practical responsibility to address this issue.^66^

 In 2015, the United Nations adopted an Agenda for sustainable development goals, ‘*Transforming the world: the 2030 Agenda for Sustainable Development*,’with 17 development goals and 169 targets. The goals are inseparable and balance the three dimensions of sustainable development: economic, social, and environmental, created to motivate action in areas of utmost importance for humanity and the planet. According to this, CE’s importance and healthcare applicability are included in at least five of the seventeen sustainable development goals (Goal 3 ‘Good health and well-being,’ ‘sustainable management of water and sanitation for all,’ 7 ‘sustainable and modern energy for all,’ ‘sustainable consumption and production patterns,’ and 13 ‘Climate action’).^57^ The most urgent area for action is climate change.^67^

 Carbon footprint, global warming and environmental sustainability are major issues with potentially effects on public health and future generations.^18^ The consequences and the significance of climate and carbon footprint in healthcare are indisputable, but strategies to reduce this impact are challenging. However, the healthcare industry is itself a significant contributor to the GHG emissions and damage to the environment.^15,17,24,26^ Most of the global healthcare GHG originates in the supply chain, making it the area of highest impact for healthcare decarbonisation. As this industry has been steadily increasing single-use disposable medical devices, emblematic of a take-make-waste economy, this has resulted in increased waste and pollution as well as associated public health damage.^68^

 The healthcare supply chain includes medical devices but also pharmaceuticals, resulting in emissions originate from materials as well as production and distribution operations. Clinicians have a major role in choosing lower emissions supplies, such as reusable medical devices rather than disposable ones and reducing unnecessary use of supplies and treatments in their practice. A minority of clinical practice is no value care — also known as overdiagnosis, the detection of harmless conditions that could be safely left undiagnosed and untreated — an example, 500 000 people a year are overdiagnosed with thyroid cancer globally and thus submitted to treatment which will not benefit them.^69,70^

 The negative consequences have been clearly highlighted: 20% of hospital admissions among over 65 are the consequence of adverse effects of prescribed drugs.

 In addition, overprescribing is identified as a major contributor to the carbon footprint; for example, it is estimated that 25%of the UK National Health Service’ (NHS’s) carbon footprint comes from medicines.^71^

 The concept of “planetary health” has also stressed the need to enhance global public health while at the same time protecting the natural systems on which humanity depends.^72^

 Within this context and along with environmental sustainability, it is necessary to assess the negative environmental impact of increased expenditure in healthcare. Nansai et al have shown that the total carbon footprint had increased by 15% in four years, with emissions resulting from patients over 65 accounting for more than half of total healthcare emissions.^50^ Malik et al highlighted the need to assess the environmental impact of these expenditures showing that the carbon footprint of healthcare accounted for 7% of the country’s GHG emissions.^47^ For the United Kingdom and Canada in 2015, it was 5% in 2015.^49^

 The climate and carbon footprint from healthcare is variable in several countries. In 2019, the healthcare climate footprint in Portugal and United Kingdom was 4.8% and 5.4%, respectively.^11^ The carbon footprint attributed to healthcare in Canada was 4.6% (2009-2015), 4.6% in Japan in 2011, 7% in Austria in 2014, 10% in the United States in 2016, 7% in Australia in 2018, and 2.7% in 2019 of China’s total GHG emissions.^46-51^ Moreover, the article published by Pichler et al, examining all OECD countries plus India and China, shows that in these countries, healthcare on average accounts for 5% of the national carbon footprint, which is comparable to the food sector. Medical retail, hospitals and ambulatory healthcare services, on average, contribute 80% of the healthcare carbon footprint. Therefore, by applying CE concepts, such as minimisation of resources use, reuse, recycling, reduction of waste, the carbon footprint of the healthcare system may be reduced. Additionally, 38% of carbon dioxide emissions are mostly associated with the heat, water and electricity generation sectors and 22% with the transport sector.^12^

 Another important aspect concerns differences in healthcare systems in high- and low-income countries and the challenges related to their environmental impact. In the latter, healthcare is insufficient, and population health is low, but it has been demonstrated that the origin of environmental impact is distributed differently between onsite contributions and supply chains in low-income countries compared with high-income countries.^66^

 The Lancet Commission on Climate and Health appealed to the health community to address “the greatest global health opportunity of the XXI century,” recommending the empowerment of health professionals to take a leadership role in investigating the environmental impact of healthcare activities.

## Application of Circular Economy Within Hospitals

 When referring to sustainability in hospitals, and referring to the search algorithm by McGain and Naylor,^15^ common research themes include hospital design, hospital building, energy, water, travel, procurements, waste, green team, staff behaviour, and telemedicine.

 The WHO stated the objective for sustainable healthcare facilities as: “All healthcare facilities and services are environmentally sustainable: using safely managed water and sanitation services and clean energy; sustainably managing their waste and procuring goods in a sustainable manner; are resilient to extreme weather events; and capable of protecting the health, safety and security of the health workforce.”^73^ In a report focusing on climate resilience and sustainability of healthcare facilities, the WHO published a comprehensive list of interventions to achieve sustainability across four dimensions: (1) health workforce, (2) water, sanitation and waste, (3) energy, and (4) infrastructure, technology and products.^57^ All of these dimensions are relevant to implement CE in healthcare and will be discussed in detail below.

###  Hospital Design

 Sustainable architecture in healthcare has been the subject of extensive research in this field, with detailed methods towards improving all aspects of hospital design, including construction, operation and maintenance.^15,37,38,46,47^ Investing in hospital design is one of the examples given by the CEO of the NHS as a tangible action that can be taken.^66^ With the initial financial expenses of a hospital building representing less than 10% of all lifetime costs.^15,19^

 The material choice/manufacture and installation phase generates the greatest carbon footprint, followed by the workforce, materials transport, roofs and masonry.^36^ Therefore, applying CE principles will contribute to a more sustainable healthcare system. For example, choice of materials that include a larger percentage of recycled materials, or the use of efficient processes in the construction phase that minimise time wasted by workers (reduces the number of visits, reducing transportation emissions for example).

 Other important features include landscaping, hospital location, using local building materials and optimising day-light and natural ventilation.^20,21,74^

###  Green Team

 The CE implementation among healthcare facilities benefits from establishing a ‘green team’ to develop interventions to achieve sustainability.^20,22-24,30^ The process starts by defining the baseline, define short- and long-term interventions, apply the intervention plan and evaluate improvements to inform the following interventions.^57^

 The “green team” also trains hospital staff regarding behaviour, awareness, correct implementation of CE protocols, and other sustainability principles. They will be responsible for designing and creating healthcare pro-environmental courses, ensuring the implementation of strategies from the perspective of CE, and reporting the environmental performance of the organisation. They should acknowledge their role in establishing a safe work environment.^20-22,30,33^

 According to the WHO, creating the “green team” is the first step in achieving sustainability, but it requires support from the top management. Local and national authorities and other external actors may also be crucial to its success.^57^

###  Waste Management

 The rapid pace of the growing global population and their use of healthcare facilities increases the waste produced, enhancing the problems in waste management. These problems are aggravated by the advances in biomedical technology, increased use of disposable medical materials and devices and is an even greater problem in low- and low-middle-income countries.^16,31,33,34^ The COVID-19 pandemic aggravated this issue due to an increase in single-use masks, gloves and other personal protective equipment used.^75^

 Waste minimisation, segregation of several types of waste and recycling programmes can have a financial and environmental positive impact, since they allow the re-introduction of these materials back into the (circular) economy.^18,20,22,27,29,32^ The financial benefit will allow an increase in the healthcare service provision, while the environmental benefit will improve the population’s health in general.

 On the contrary, the absence of effective activities and low levels of training and awareness for healthcare waste management could increase the spread of diseases. Consequences encompass decreased quality of healthcare provided and the probability of infection/security of the staff handling of this waste. This could also result in public health risks during waste transportation and regarding contamination of underground aquifers through the unprocessed medical waste placed in landfills.^34^

 According to the WHO about 85% of a hospital’s waste is general, non-hazardous waste, with only 15% being hazardous material (infectious, toxic or radioactive).^76^ Therefore, most hospital materials are recyclable, leading to environmental and financial savings.^34^ Waste segregation into different categories will reduce hazardous residues and the costs of waste streams^22,33^ and reduce the impact on carbon footprint,^77^ requiring adequate staff training.

 Even though hazardous waste represents a small fraction of all waste produced, its management can represent health, safety and environmental risks making it necessary to enforce strict control of their disposal.^30^ The infectious healthcare waste requires pre-treatment before being recycled and reused due to its hazardous nature.^30^ Regarding healthcare solid waste management, composting and recycling are the best solutions, since the materials are given a second life, while landfilling and incineration are the worst.^29,35^

 Identification, quantification and reporting of hospital waste production are essential. This includes evidence of waste composition, identifying wasteful practices, and determining measures to handle these specific waste materials or components, providing feedback on waste segregation and minimisation efficiency.^22,33^ A reduction in healthcare waste can be achieved by choosing medical materials and devices of low hazardous trace^22,33^ and through the replacement of single-use medical devices with reusable ones.^77,78^ LCA allows for the assessment of environmental and financial costs and can be used to design reusable products to replace the disposable ones (an example would be sterilisation of such products as an alternative to incineration).^22,33,34^

 Legal and economic strategies could be enforced for manufacturers of medical supplies to comply with CE principles. Examples include fiscal benefits to those producing highly reused goods with low hazardous compounds, minimising possible manufacturing-specific waste. Such strategies would aim to enhance the circularity of products.^34^ This will result in the manufacturers being held accountable for the disposal and recovery costs, and at the same time, providing them with major encouragement for implementing recycling protocols.^1^

###  Energy

 Energy consumption is part of the CE concept since one of its principles is the reduction of resource extraction. Energy is one of these resources, either directly, or indirectly by referring to the resources needed to produce this energy (fuel, renewable energy equipment, etc).

 Standard operating procedures for hospitals require significant energy use, with heating, ventilation and air conditioning accounting for 50% of direct energy costs, with lightning and equipment accounting for the remainder.^29^ The lack of energy directly affects healthcare facilities functioning, leading to the malfunction of medical equipment, from refrigerators to diagnostic and medical devices,^57^ resulting in an aggravation of patients’ health. The energy use in healthcare facilities must be a good balance between proper functioning and waste.

 Relevant measures to improve energy efficiency may include replacing halogen light with LED, using alternative forms of clean and renewable energy, solar and wind energy and biofuels, thermal insulation, self-closing doors, pump and boiler house upgrades, heat recovery on air handling units, the subdivision of heating circuits, electric car charge points and renewable energy options, switching off equipment and lights when not in use, and many more strategies.^19,21,24,25,57^ If possible, renewable energy onsite ensures a more reliable and flexible operation, making healthcare facilities more resilient to extreme weather events.^23,57^ Although initially considered as an expenditure associated with the use of resources in its manufacturing, the use of alternative energy will provide significant financial and material savings in the future.^15^

###  Water

 Water is a resource that is extremely scarce in many areas of the world, and its use should be minimised to the essentials worldwide. Climate changes aggravate this issue and will further worsen the situation. Hospitals consume considerable amounts of water — eg, 1% of a city’s total water consumption.^21^ This can be mitigated through efficient water use, such as applying flow restrictors, checking for leaks, and proper equipment maintenance. Hospitals can collect up to 85% of rainwater and recycle water for non-potable purposes.^18,19,21,25^ The principles of CE applied here are two: reduction of resource use and reuse/recycling of water.

 Water resources should be protected as much as possible within healthcare facilities because water is essential for the services provided within hospitals and clinics, including for sanitation and hygiene, which will result in an increase of food-, vector- and water-borne diseases.^57^

 Additionally, water consumption is associated with other sustainability issues. Avoiding bottled water should be promoted to minimise plastic pollution and unnecessary use of resources,^23^ and the use of energy to heat, pump and dispose of water is also associated with the issue of energy consumption.

###  Travel/Transportation/Telemedicine

 GHG emissions from travel and transportation represent 27% of total EU GHG emissions,^79^ and 16% related to healthcare staff/patient travel.^15^ These are associated with CE concepts since human resources use additional resources such as transportation that require other resources (such as fuel, tyres, materials for manufacturing). Efficient use of human resources, transportation modes, and fuels (including renewable energies) may greatly impact the reduction in resource use.

 Healthcare-related GHG emissions can be reduced through the use of bicycles, public transportation and alternative-fuel vehicles by patients and staff. Since this depends solely on their willingness to do so, raising awareness is imperative.^15,23,45,74^

 The use of telemedicine might also reduce staff and patient travel-related emissions.^39-45^ The increase in telemedicine during the COVID-19 pandemic showed that this is a viable solution.^75^

 Another important issue is the transportation of food (discussed further below) and healthcare waste. Reduction of travel distance by choosing closer waste treatment centres and buying from local suppliers or from suppliers using environmental-friendly transportation leads to a reduction of fossil fuel use and GHG emissions.^29,74^

###  Procurement

 CE has a direct impact on the goods produced and procured. If goods are produced in a CE, there is a reduction in resources extracted from nature and an increase in the re-processing of waste. Procured goods also are the main contributor to healthcare’s carbon footprint since GHG are emitted both in their production and transportation.^16,17^

 Sustainable procurement/purchasing can thus reduce GHG emissions at healthcare facilities and could be implemented through the hospital purchasers to influence their suppliers, ensuring that these goods and services are produced sustainably.

 Procurement should include purchase criteria that favours environmental friendly products, local suppliers/supplies, reduce and reuse packaging as much as possible and acquisition of durable equipment/devices to increase product life.^12,19,20^ The LCA method could allow hospitals to better understand the environmental and financial costs of items or processes purchased.^15,26^

 Over the past few years, hospitals have been using considerable amounts of disposable items, with many reusable products being replaced by disposable ones. For example, a significant reduction of the sharps waste stream’s carbon footprint has been obtained through purchasing reusable sharp containers instead of disposable ones.^77,78^

 The extinction of materials will directly affect healthcare services since diagnostic and therapeutic devices need specific materials to be produced.^57^ As such, healthcare should pioneer the implementation of CE practices to reduce, reuse and recycle rare/overused materials.

###  Food 

 Hospital food services can negatively affect the environment at every stage of the food supply chain (production/procurement, distribution, preparation, consumption, and waste management/disposal).^80^

 Promoting and supporting sustainably grown local food for staff and patients would provide healthier nutrition and reduce transport between farmers and the hospital, reducing the hospital climate footprint.^18,19,21,23-25,74^ In other words, there would be a reduction on the use of resources (R - Reduce) in their transportation.

 Other measures include: reusing unserved food where appropriate, providing patient menus with portion size, limiting the amount of food served to the person’s needs (R - Reduce), use leftovers in the preparation of new dishes in a safe manner (R - Reuse), and composting food waste (R - Recycle), introduce an onsite garden.^33,19^ Food can also be served in different manners; avoiding disposable items, and replacing them with reusable utensils, is more beneficial for the environment, reducing waste and the production of new items that require new resources.^21^

###  Behaviour

 As discussed before, many of the strategies depend on individuals’ awareness and willingness to apply these strategies.^57^ Such as bringing a refillable water bottle (instead of single-use plastic), composting non-reusable food, close the water tap when not in use, among others. The individuals within an organisation are also the drivers that may implement new practices that affect healthcare’s sustainability.

 A recent survey by The Economist showed that healthcare professionals are deeply concerned about the growing impact of climate change and eager to see sustainability rise on the agenda in their workplace and that they might be more proactive if they had more support and incentives.^81^

 Staff and patients have a major role in GHG emissions reduction strategies in hospitals.^16,78^ Such changes depend on greater awareness towards waste reduction, segregation, recycling, efficient use of water and energy. It is essential to address these issues to foster and engage sustainable practices and awareness among hospitals users and workers, regarding their view of hospitals/healthcare sustainability.^15,17,18,20,25,27,28,31,82^

## Discussion

 Climate change represents a global threat. This is expected to continue to change over this century, requiring concerted actions to reduce the use of natural resources, reuse and recycle used materials, and reduce GHG emissions and subsequently the carbon footprint. These are a product of our ‘take-make-dispose’ economy, which relies on fossil fuels and does not manage resources for the long-term.^83^

 Healthcare facilities are a major contributor and have a major role in reducing their impact on natural resources use and GHG emissions. However, we are still at the beginning of this path and much more needs to be done with regard to healthcare climate footprint. The 2019 report from the United Nations on ‘*The Sustainable Development Goals Report*’ shows that, although some advances were made, monumental challenges still remain. Evidence and data highlight areas that require urgent attention and more rapid progress to accomplish the 2030 Agenda’s far-reaching vision. Despite the mentioned goals of good health and well-being, as well as climate action, the 2030 Agenda shows no specific goals or priorities for hospital involvement in environmental sustainability. Moreover, due to their impact on carbon footprint, it is of paramount importance to consider hospitals in the core of climate change mitigation.

 This literature review showed that the EU, at central and national levels, believes in the CE as a central mechanism to achieve the goals of the Paris Agreement.^60,61^ Several actions were introduced to promote CE and GHG reduction, aiming to improve sustainability and promote growth and job creation.^3,55,56^ Therefore, the EU aims to achieve an economically sound sustainability. The literature seems to show that CE implementation in the EU is still at the beginning of its potential. Most measures focus on recycling, with some secondary materials showing some potential against virgin material,^1,5,7^ but other dimensions of CE are starting to be implemented: material-efficient products; sustainable production; environmental technology verification; ecolabel, green public procurement, eco-design and energy labelling; best environmental management practice; and sustainable use of resources (water, soil, raw materials, and minerals); energy efficiency and renewable energy.^5,59^ The literature did not offer insight into the current application of CE in healthcare at the European level.

 From the literature review, few articles on the applicability and implementation of CE in healthcare and specifically in hospitals were found, while the concepts are often narrowly studied, and rarely implemented in hospitals as a whole (Table 2). With respect to the evaluation of healthcare GHG emission or carbon footprint it is restricted to the United States, Australia, China, Canada, Austria, and Japan (Table 3). With regard to hospitals, in this review, only 12 articles present an overview of sustainability applicability within hospitals, namely scope 1, in line with the seven elements of climate-friendly hospitals from the WHO and Health Care Without Harm. The remaining nineteen referred to specific themes within hospital sustainability, namely scope 2 (waste), 3 (green buildings) and 4 (transportation/telemedicine) (Table 2). Also, a small number of publications (seven) referred to hospitals as significant contributors to resource depletion and therefore to carbon footprint as well as to environmental change/environmental sustainability.^15-18,23,24,28^

 A significant number of publications about environmental sustainability within hospitals were from the United Kingdom (eight) and Europe (eight), followed by the United States (six), perhaps because they are more committed to carbon neutrality or have more resources available. The United Kingdom created the Sustainable Development Unit in 2008 and the ‘Greener NHS programme,’ showing their commitment to be the world’s first net-zero health service, improving health for present and future generations. This engagement also comes from the EU to respond to climate changes and to achieve carbon neutrality by 2050. The US health service is based on private insurance, and it is the country with the most significant GHG emissions, so possibly there are many economic concerns (Table 3).

 At the EU level, it is important to define the sectors, which in this context is healthcare, and the organisations within, namely hospitals, which can fall within the scope of the CE. The current CE approach to hospitals is very restrictive, because its focus is limited to waste management, mainly due to European and individual country policies. Furthermore, regarding the literature, waste management is one of the most studied subjects (scope 2), alongside energy and followed by travel/transportation/telemedicine, with potential benefits for fostering environmental sustainability in hospitals (Table 2).

 Despite healthcare waste management being one of the most studied subjects regarding the sustainability within hospitals, this area faces many challenges.^30^ An appropriate healthcare waste management system should contribute to improve the segregation of healthcare waste, reducing the environmental impact of hospital and health risks, towards the improvement of the quality of life of the population.^30,34^ Another point that should be highlighted is that healthcare waste management comprises several streams, and some components of healthcare waste cannot be reused, recycled or composted.^1,22^ Hospital waste is constituted by many different types of waste, creating a challenge to its management.^33^ To resolve this issue, an overall assessment of the current healthcare waste management is fundamental. This can help identify gaps and prioritise available options. These options can be standardised and used for subsequent monitoring and evaluation. Adopting a CE approach to these streams as a whole is not possible; therefore, extended producer and consumer accountability needs to be implemented. In healthcare infectious waste, the current linear solutions still need to be used, since it is not possible to create value from these materials.^1,22,33^ This challenge was especially evident during the current COVID-19 pandemic because of the increased volume and infectious nature of the waste generated.^75,84,85^

 Other themes referring to CE and sustainability in general applied to hospitals included: ‘Green team,’ green procurement, green building (energy efficiency and construction), food (sustainable production and transportation), engagement and environmental behaviour.

 The role of hospitals is not limited to their own direct practices. When sustainability is studied in the context of healthcare, the focus is often on direct energy consumption by hospitals/clinics, while ignoring the energy consumption of during manufacturing of goods used and their transportation. The indirect impact caused by staff and clients (patients) is also relevant and must be addressed. Narrow views like this should be expanded to include both direct and indirect resources use, waste management, energy and water use and carbon emissions.

 Healthcare sector has the opportunity to be involved in climate change action in several areas. The implementation of CE in healthcare should follow several steps connected with the sustainability of hospitals where progress has been previously achieved. All steps need to include a ‘Green team’ creation, reduction and conservation of resources (including energy and water); maintenance of these systems; use of clean energies; construction of more efficient buildings (reducing resources use) and with local materials (reducing resources use in transportation); stimulating green procurement; encouraging staff/patients to use more eco-friendly means of transport; promoting and supporting sustainable and conscious grown local food and investment in waste management as well as engagement of all stakeholders in more sustainable and environmental behaviours in CE daily practices.

 Almost every article identified the need for engagement of individuals and sustainable/environmental behaviour for a full application of environmental sustainability practices (Table 2). This includes to involve staff and patients, given their role in the hospital’s environmental impact. Staff awareness has been identified as one of the most important factors required for hospital improvement since they are the drivers of change within the hospital.^15,33^ Additionally, some literature refers the importance of proper waste management training for hospital staff in a successful implementation.^28,30^

 From this perspective, healthcare has a major role in reducing resources use, reuse and recycling, including a reduction in GHG emissions, minimising emissions as low as reasonably achievable, without forgetting the main goal as a healthcare provider. In this regard, the CE concept brings new tools to develop and implement within healthcare and more particularly, in the hospital setting. Additionally, it is also crucial that healthcare stakeholders raise awareness of this problem across other businesses, given the impact of pollution and other environmental issues on the population’s health.

 This overview regarding CE implementation and applicability in hospitals practice was presented to provide a comprehensive picture of the actual situation and help to identify how CE can be implemented in current hospitals. As addressed before, the concept of CE was applied to this study in its wider scope, since it addressed not only the reduction in natural resources extraction, the reuse of resources extracted and the re-introduction of resources back into the economy, instead of becoming waste; but also interprets the use of energy and water as resources, and addressed GHG emissions as part of the CE since they are emitted as a result of the use of fossil fuels, which are a resource.

###  Recommendations for Future Research

 The literature identified specific areas and behaviours which hospitals can address to improve their use of resources. Therefore, more research is needed to improve the implementation of CE in specific areas of healthcare services. The number of articles regarding its implementation in hospitals is limited and the literature is scarce regarding the adoption, applicability and implementation of CE practices in hospitals. It is important to start with analysing the actual scenario of hospitals and implement studies of CE practices in the ground in order to collect data to create guidelines/recommendations as well as to inform government and encourage national policies. This literature review also highlighted the need for public health measures and policies to reduce the environmental impact of healthcare.

 The lack of staff awareness and knowledge of the environmental impact of healthcare and hospitals’ sustainability were identified as major contributors to the issue, yet, there is scant literature discussing green skills of healthcare professionals. For example, the influence of psychological and social factors in the use of resources, the transport options and the interaction with the building and technologies available.

 This is particularly important as a commitment of the clinical community is imperative. We have learnt from the COVID-19 pandemic that the union of these professionals through shared information and innovative models of care could successfully overtake limited resources and inequities in access to care.^69^

###  Limitations

 This research is subject to some limitations. The first is the lack of previous studies on the topic dedicated to healthcare and hospital environment, bringing in the need of initial articles classification into scopes to obtain an overview of the existing research, which could be unclear. It became clear that there is no specific research about CE in healthcare, and therefore, this literature review addresses the sub-components of CE: resources use and reuse (including water, energy, food and other unconventional resources), recycling and re-introduction of resources back into the economy, and a reduction in GHG.

 Also, the search query, which was specific to the healthcare sector, could miss relevant literature studying other sectors, but which strategies could apply to healthcare. However, having an open review of the literature regarding CE without specifying the sector would create an unbearable amount of literature with questionable application to healthcare.

 Another limitation that could be addressed in future research is the absence of quantitative analysis, which could advise policy-makers and hospital managers regarding CE policies — furthermore, the need for research regarding the relationship between healthcare CE practices and hospital sustainability.

 The scope defined could be starting point areas for introducing CE at hospitals, but methodologies of process introduction evaluation were not identified. Besides, the behaviour and engagement of healthcare professionals with CE practices, methods to incentivise hospital managers to introduce these practices and to incentivise the workforce to apply them need to be studied more.

 Recommendations of CE policies were not defined for hospital administrators along with the daily management of these policies in the routine of hospitals.

## Conclusions

 In conclusion, all the highlighted themes that emerged from this review are within CE scope, and have a huge applicability and could potentially improve hospitals’ sustainability. As such, this review can assist decision-makers on the way forward to implement environmentally-friendly CE practices in current hospitals. Therefore, the main recommendation is that healthcare facilities evaluate their application of CE practices and other sustainability strategies in the different domains identified in this study: waste management, energy, water, travel/transportation/telemedicine, procurement, food and behaviour.

 Considering that the notion of CE has attracted increased attention in recent years, an integrated, multisectoral, political approach is required for addressing persistent, systemic environmental changes. A sustainable future will demand that organisations, which in the health sector would specifically include hospitals, adopt systems that prioritise the three pillars of sustainability: society, environment and economy. Re-thinking these issues, should lead to more balanced societies in equilibrium with the rest of the biosphere. From this perspective, awareness and knowledge regarding proper CE practices in hospitals are of paramount importance.

## Ethical issues

 Not applicable.

## Competing interests

 Authors declare that they have no competing interests.

## Funding

 This work was co-funded by the SAFE EUROPE project under the Erasmus+ Sector Skill Alliances programme [grant agreement 2018e2993/001-001]. The European Commission support for the production of this publication does not constitute an endorsement of the contents which reflects the views only of the authors, and the Commission cannot be held responsible for any use which may be made of the information contained therein.
